# Cost-effectiveness analysis of oral nutritional supplements with nutritional counselling in head and neck cancer patients undergoing radiotherapy

**DOI:** 10.1186/s12962-021-00291-7

**Published:** 2021-06-15

**Authors:** Beatrice Martin, Emanuele Cereda, Riccardo Caccialanza, Paolo Pedrazzoli, Rosanna Tarricone, Oriana Ciani

**Affiliations:** 1 Department of Social and Political Science, Bocconi University, Milan, Italy; 2 Clinical Nutrition and Dietetics Unit, Fondazione IRCCS, Policlinico San Matteo, Pavia, Italy; 3 Medical Oncology, Fondazione IRCCS, Policlinico San Matteo, Pavia, Italy; 4 Department of Internal Medicine and Medical Therapy, University of Pavia, Pavia, Italy; 5 SDA Bocconi School of Management, Centre for Research On Health and Social Care Management (CERGAS), Milan, Italy; 6 College of Medicine and Health, University of Exeter, Exeter, UK

## Abstract

**Objective:**

There is limited evidence regarding the economic effects of nutrition support in cancer patients. This study aims at investigating the cost-effectiveness profile of systematic oral nutritional supplementation (ONS) in head and neck cancer (HNC) patients undergoing radiotherapy (RT) and receiving nutritional counseling.

**Methods:**

A cost-effectiveness analysis based on a RCT was performed to estimate direct medical costs, life years gained (LYG) and Quality-Adjusted Life Years (QALY) for nutritional counseling with or without ONS at 5-month and 6-year follow up time. Value of information analysis was performed to value the expected gain from reducing uncertainty through further data collection.

**Results:**

ONS with nutritional counseling produced higher QALY than nutritional counseling alone (0.291 ± 0.087 vs 0.288 ± 0.087), however the difference was not significant (0.0027, P = 0.84). Mean costs were €987.60 vs €996.09, respectively in the treatment and control group (-€8.96, P = 0.98). The Incremental Cost Effectiveness Ratio (ICER) was -€3,277/QALY, with 55.4% probabilities of being cost-effective at a cost-effectiveness threshold of €30,000/QALY. The Expected Incremental Benefit was €95.16 and the Population Expected Value of Perfect Information was €8.6 million, implying that additional research is likely to be worthwhile. At a median 6-year follow up, the treatment group had a significantly better survival rate when adjusting for late effect (P = 0.039).

**Conclusion:**

Our findings provide the first evidence to inform decisions about funding and reimbursement of ONS in combination with nutritional counseling in HNC patients undergoing RT. ONS may improve quality of cancer care at no additional costs, however further research on the cost-effectiveness of nutritional supplementation is recommended.

*Trial Registration*: ClinicalTrials.gov: NCT02055833. Registered 5th February 2014 https://clinicaltrials.gov/ct2/show/NCT02055833

**Supplementary Information:**

The online version contains supplementary material available at 10.1186/s12962-021-00291-7.

## Highlights

i. What is already known about the topic?
  It has been shown through a randomized controlled trial that counseling coupled with oral nutritional supplements (ONS) in head and neck cancer (HNC) patients undergoing radiotherapy results in smaller loss of body weight, higher protein-calorie intake, improvement in quality of life over time and reduced need for radiotherapy (RT) and/or systemic therapy (ST) dose reduction or complete suspension than nutritional counseling alone
ii. What does the paper add to existing knowledge?
  This paper investigates the cost-effectiveness of ONS in combination with nutritional counseling in HNC patients using individual patient data from a recent RCT at 5-month and 6-year follow up time. This paper also quantifies the value of conducting further data collection in this field.
iii. What insights does the paper provide for informing healthcare-related decision making?
  This study shows that neither differences in QALY (0.0027, P = 0.84) nor costs (-€8.96, P = 0.98) were statistically significant. ONS has 55.4% probability of being cost-effective at a cost-effectiveness threshold of €30,000/QALY at 5 months. Population Expected Value of Perfect Information was €8.6 million, implying that additional research is likely to be worthwhile. At a median 6-year follow up, the treatment group showed a significantly better survival rate when adjusting for late effect (P = 0.039), and a corresponding ICER of -€31.97/LYG.

## Introduction

Malnutrition is a common clinical and public health problem that afflicts individuals both in community and hospital setting [1, 2]. Rates above 30% have been reported among hospitalized patients and among residents in Rehabilitation Institutions and Nursing Homes in Italy [3, 4]. Malnutrition is known to delay recovery from illness, to increase complications and dependency on others, to deteriorate quality of life and to extend length of stay [3]. Treating malnutrition has become an integral part of medical treatment and prevention. Whilst the general benefits of nutritional support on clinical outcomes are well recognized, there is much less information about its economic consequences.

Head and neck cancer (HNC) patients are at high risk of malnutrition [4–7], which has a negative effect on different clinical outcomes, including an impaired quality of life and a worse overall prognosis [6, 8]. Many cancer patients develop treatment-related toxicities that can cause or exacerbate symptoms, such as mucositis, xerostomia, alteration or loss of taste, fatigue, nausea and vomiting, which further increase the risk of malnutrition [9–11]. As a consequence of such toxicities, patients may suspend or reduce radiotherapy (RT) or systemic therapy (ST), which may cause poor clinical outcomes [9, 12, 13].

Early nutritional intervention with high protein-calorie intakes has been associated with clinical, nutritional and functional improvements and significant reductions in the number of hospital readmissions [14–17]. Cereda et al. [18] found that counseling coupled with oral nutritional supplements (ONS) resulted in smaller loss of body weight than nutritional counseling alone (mean difference, 1.6 kg, P = 0.006), higher protein-calorie intake and improvement in quality of life over time (P < 0.001 for all). Moreover, the use of ONS reduced the need for RT and/or ST dose reduction or complete suspension (P = 0.029). This favorable clinical profile [18] could be coupled with a favorable economic impact in a disease area, such as cancer, where health spending is increasing faster than the increase in cancer incidence [19]. The diffusion of innovation in this therapeutic area is progressively improving survival for cancer patients [20, 21], however imposing further constraints on available resources already scarce. The need for economic evaluations becomes even stronger to inform decisions about which interventions to prioritize and how to ensure maximum health benefit is gained out of fixed budgets. In the example of nutritional supplementation, there is scant evidence regarding its cost-effectiveness profile, at a time when, given the uptake in clinical practice, national or regional decision-making bodies may want to decide on whether this intervention should be covered for the relevant patient population. Therefore, the aim of this study was to investigate the cost-effectiveness of ONS provision in combination with nutritional counseling in HNC patients using individual patient data from a recent randomized controlled trial.

## Methods

This trial-based cost-effectiveness analysis is reported according to the Consolidated Health Economic Evaluation Reporting Standards (CHEERS) statement [22]. The horizon for the analysis matched the follow-up period for the underlying clinical trial (5 months) and updated survival status check up to 8 years post-randomization.

### Randomized controlled trial

A prospective single-center RCT (ClinicalTrials.gov: NCT02055833) on nutritional counseling with (N = 78) or without (N = 81) systematic use of ONS in HNC patients undergoing radiotherapy has been extensively described [18]. Adult patients with newly diagnosed HNC, candidate for RT or RT plus ST were recruited and randomized from the start of RT, lasting 2 months, and for up to 3 months after its end. Tolerance to cancer treatments was continuously monitored. Compliance in the ONS arm was assessed by caregivers and dietitians recording with a diary the number of bottles consumed every day and was on average equivalent to 1.2 bottles/day (SD = 0.6). If patients were unable to maintain satisfactory oral intakes, enteral or parenteral nutrition support was started.

### Utility weight

Health-related quality of life (HRQoL) was assessed in the clinical trial using the European Organization for the Research and Treatment of Cancer Quality of Life Questionnaire (EORTC QLQ-C30) at baseline, at the end of RT, 1 and 3 months after the end of RT. Since EORTC QLQ-C30 do not incorporate preferences, they were translated into EQ-5D derived health state utilities using a mapping algorithm [23]. The algorithm used data from patients with HNC, the beta regression model showed best model fit (R^2^ = 0.39, MAE = 0.0949, RMSE = 0.1209) with global health status, physical, role and emotional functioning and pain scales as predictors. HRQoL data were not available for dropout patients (due to death, hospitalization, artificial nutrition, loss to follow up and withdrawal for unknown reasons) and 8 additional single data points from 7 patients. A literature search was conducted to assign missing health utilities according to reason of dropout (hospitalization, need of artificial nutrition during or after RT) (Table 1). Chained multiple imputation [24] was performed for the remainder of cases. Independent variables for the imputation were age, gender, diagnosis of malnutrition at inclusion, tumor site and stage, handgrip strength and phase angle [18]. Linear Mixed Models (LMM) with random intercepts to account for the lack of independence of repeated measures was used to assess the difference over time in utility weights among treated and control groups. Health outcomes in our analysis were expressed in terms of quality-adjusted life-year (QALY), a metric that accounts for both longevity and quality of life. We estimated QALYs accrued for each subject as the interpolated area under the utility weight curve. A comparison of average QALYs gained among the two groups was performed through t-test and bootstrap percentile method.

**Table 1:** Unit costs and other parameters for the cost-effectiveness analysis

Variable	Value	Unit Cost (€)	Year	Source
ONS treatment (bottle)		3.23	2017	Local healthcare system, Pavia
Service ONS treatment (day)		2.26	2017	Local healthcare system, Pavia
ONS control (bottle)		1.43	2017	Local healthcare system, Pavia
First medical visit		28.50	2017	Local healthcare system, Pavia
Follow-up medical visits		17.90	2017	Local healthcare system, Pavia
Service EN (day)		6.18	2017	Local healthcare system, Pavia
Mean compound EN (day)		4.20	2017	Local healthcare system, Pavia
Service + bag + nursing PN (day)		43.40	2017	Local healthcare system, Pavia
Severe Mucositis		45.00	2008	Banz [51]
Hospitalization		608.00	2004	Ministero dell’Economia e delle Finanze^a^ (2007)
Utility values imputed				
Hospitalization and artificial nutrition before end of radiotherapy	0.35			Kim [50]^b^
Hospitalization and artificial nutrition after end of radiotherapy	0.30			Kim [50]^c^

*EN* Enteral Nutrition, *PN* Parenteral Nutrition

^a^Ministero dell’Economia e delle Finanze Commissione Tecnica per la Finanza Pubblica (2007)-page 43

^b^Acute and late toxicities from chemotherapy

^c^Acute and late toxicities

### Costs

Only direct medical costs were accounted for under the perspective of the Italian National Health System (NHS). The costs included were decided following a discussion with clinical experts (Table 1). All costs were adjusted for inflation to obtain 2017 equivalents. Data about healthcare resource consumption were collected at each follow-up visit, no missing data imputation was performed in terms of resource consumption. To investigate the difference in mean costs among the two arms, t-test as well as bootstrap percentile method to deal with highly skewed distributions and heavy right tails were used.

### Cost-effectiveness analysis

We report the results of the cost-effectiveness analysis in terms of cost per QALY saved. Although there is no official cost-effectiveness threshold in Italy, the commonly accepted threshold of €30,000 per QALY is considered [25]. A non-parametric bootstrapping re-sampling technique (N = 1000) was used to investigate the uncertainty around the incremental cost-effectiveness ratio (ICER) using the percentile method. Probability distributions were assigned to costs and outcomes in order to perform a probabilistic sensitivity analysis, to explore the reliability of the ICER in relation to the uncertainty in the inputs derived from the clinical trial. The most suitable parametric families for costs and effects in the treatment and in the control arm were identified according to Akaike information criteria (AIC) (Additional file 1: Table 1). These bootstrap results were used to build the cost-effectiveness plane (CEP), plotting the 1000 bootstrapped incremental mean costs and mean QALY pairs, the cost-effectiveness acceptability curves (CEAC), representing the probabilities that the intervention is cost effective (y-axis) against all potential values of WTP thresholds (x- axis) in the interval (0–50,000), the Expected Incremental Benefit (EIB), calculated by multiplying the number of QALYs saved by the cost-effectiveness threshold and subtracting the difference in costs and Expected Value of Perfect Information (EVPI). The per patient EVPI is defined as the difference between the value associated with a decision made on the basis of current information and the value that could be expected if perfect information were available on which a decision could be based. The incidence of HNC in Italy in 2019 has been estimated to be 9,300 patients [26]. Based on the Italian Cancer Registry 2019 [27] data and on expert opinion, we assume 60% of these patients (i.e. 5580) are candidates for RT and thus will be affected by the information yearly over a lifetime of 10 years and a discount rate of 3.5% per year, in order to derive the Population EVPI.

Finally, a cost-effectiveness analysis after a median 6-year follow-up time post-randomization, calculated using the reverse Kaplan Meier [2200 days 95% CI (1815; 2432)], was conducted exploiting updated survival data of patients in the trial. Data about survival were collected on January 2nd, 2020 and presented using Kaplan–Meier (KM) survival curves. Patients were enrolled from July 2012 to April 2016, thus subjects enrolled late in the study were treated as censored if, as of January 2nd 2020, they were still alive. To compare survival curves, two approaches were adopted: Restricted Mean Survival Times (RMST) and weighted log-rank test, which better accommodate departure from proportional hazard assumption [28]. Since no cost information was available for subjects after the end of the trial, we assumed that costs incurred after this time were independent of the patient’s group [29]. Consequently, the same incremental cost estimates from the 5-month follow up analysis were considered for the 6-year follow up analysis. In this case, results are reported as incremental costs per life year gained (LYG). LYG are calculated as the difference in mean survival at time τ (through RMST) and are discounted at 3.5% yearly rate.

## Results

### Health outcomes and QALYs

**Fig. 1:**
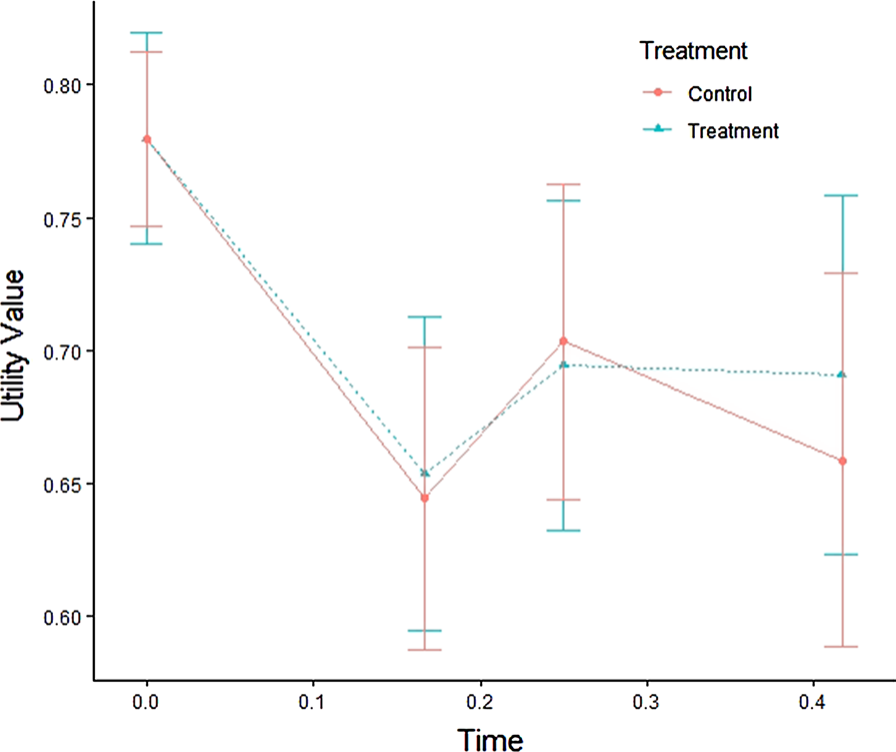
Utility profile of treatment (ONS + nutritional counseling) and control (nutritional counseling) group

In total 8 patients in the treatment arm and 7 patients in the control arm died during the 5 months of the trial. The utility profiles of treatment and control group are reported in Fig. 1. The LMM coupled with Satterthwaite’s method and the Likelihood Ratio Test comparing the likelihood of the model with and without the interaction term show that there was not a significant difference in terms of utility values over time between treatment and control (P = 0.79 for both). The mean QALY in the treatment group was 0.2911 (SD 0.0870) while the mean QALY in the control group was 0.2883 (SD 0.0866), resulting in a difference of 0.0027 in favor of the treatment group but it was not statistically significant (t-test P = 0.84; bootstrap percentile method P = 0.86).

### Costs

The mean costs in the intervention group were €987.60 ± 776.96, the mean costs in the control group were €996.09 ± 2546.88, returning a difference of -€8.96 (t-test P = 0.98; bootstrap percentile method P = 0.96). Disaggregation of cost components (Table 2) reveals that the main drivers of costs were the direct cost of intervention (“ONS + service”) in the treatment arm, while parenteral nutrition and hospitalization in the control group. The difference in mean costs was not significant from both a statistical and economic point of view, meaning that the additional costs of ONS were offset by the higher hospitalization and artificial nutrition costs in the control group.

**Table 2:** Average cost per patient by cost categories

	Treatment	Control	p-value
ONS (+ service)	673.19	50.69*	< 0.001
Medical visit	7.04	10.05	0.32
Enteral Nutrition	75.99	87.01	0.80
Parenteral Nutrition	105.16	352.56	0.06
Hospitalization	112.81	479.78	0.18
Mucositis	13.41	15.99	0.48
Total	987.60	996.09	0.98

^*^ONS were also prescribed to 8 patients allocated to control group in order to improve their protein-calorie intake

### Cost effectiveness

**Fig. 2:**
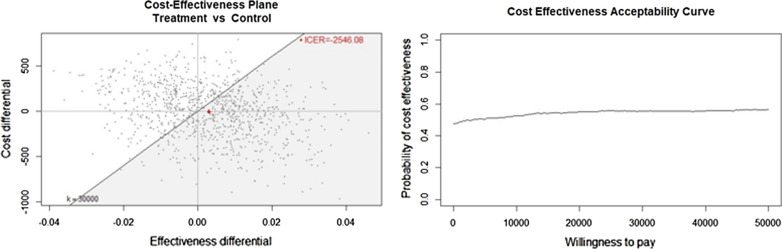
Cost Effectiveness Plane (on the left) at a WTP = €30,000/QALY and Acceptability Curve (on the right)

The resulting ICER from this randomized experiment was -€3,277/QALY (-€8.96/0.0027QALY). Since the intervention was both less costly and more effective, it is considered a dominant strategy. However, neither of the differences in QALY and costs were statistically significant, it has thus been essential to explore the uncertainty around this result through bootstrapping resampling technique. From the CEP in Fig. 2a, the intervention had 55.4% probabilities of being more cost effective than the control at a WTP of €30,000. The resulting ICER was -€2,546/QALY (-€7.44/0.0029). However, the dispersion of the pairs all over the four quadrants signals the high level of uncertainty whereby it is not possible to draw a line through the origin that excludes 2.5% of the distribution of the difference in costs and effects, meaning that the 95% confidence interval for the ICER was undefined [30]. The CEAC in Fig. 2.b is approximately stable at 50–60%, meaning that the WTP does not influence much the level of confidence on the probability of cost-effectiveness. At a WTP of €30,000 the EIB = 95.16, thus the treatment was on average more cost-effective than the control. However, the 95% confidence interval of EIB includes zero (Additional file 1: Fig. S1a), therefore we cannot affirm that it was significantly different from zero, for any value of WTP. Finally, at a WTP of €30,000, the EVPI was 184.39 per patient (Additional file 1: Fig. S1b). The population EVPI over a 10 years horizon is 8.6 million. Given that the cost of conducting further research is likely lower than €8.6 million, additional data collection is probably going to be beneficial [29, 31].

### Probabilistic sensitivity analysis

A Gamma and Weibull distribution represented the best fit for costs distributions in the treatment and control arm, respectively (Additional file 1: Table S1). The distribution of QALY in treatment and control had long, heavy left tails, ranging from 0 to 0.417. A right-skewed Weibull distribution was used to fit values’ complement to the maximum theoretical value of 0.417 (Additional file 1: Table S1). 1000-random samples from these distributions yielded an ICER of -€3,260/QALY (-€10.8/0.0033QALY) with 53.8% probability of being cost-effective at a cost-effectiveness threshold of €30,000/QALY (Additional file 1: Figure S2). The EIB at the same cost-effectiveness threshold was €110.32, indicating the ONS with nutritional counseling was on average more cost-effective than nutritional counseling alone although this cannot be stated for any value of the cost-effectiveness threshold, given the 95% confidence interval of EIB crosses zero. The EVPI slightly increases to €194.5 per patient. Overall, the results of the PSA confirm previous findings but further highlight the uncertainty in the decision, mainly related to costs.

### Cost-effectiveness analysis up to 6-year follow-up time

**Fig. 3:**
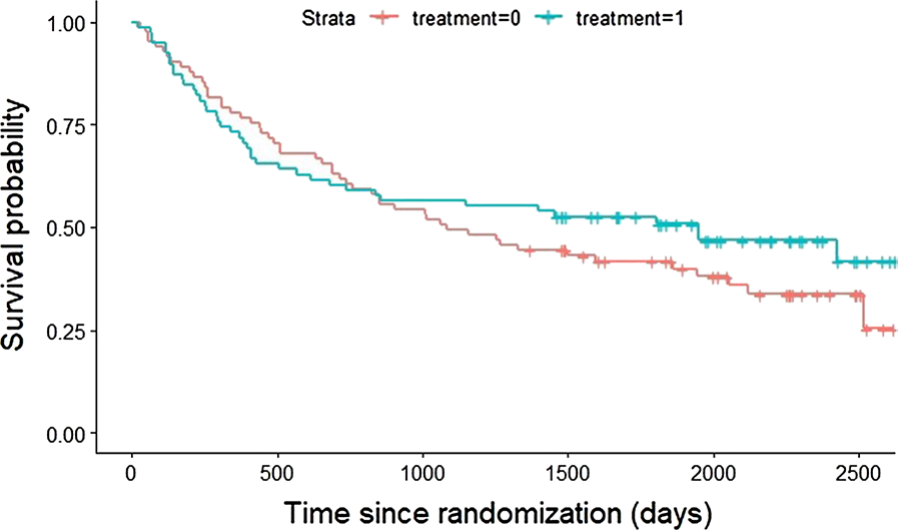
Survival curves of treatment and control group

Figure 3 reports the KM survival curves for each arm at a median follow up of 6 years since randomization. The median survival of ONS with nutritional counseling group was 1949 days (5.34 years) while median survival was 1085 days (2.97 years) for the nutritional counseling group. Table 3 shows mean Overall Survival (OS) of treatment and control patients up to 6 years calculated as the area under the two KM curves (RMST) over a specified time, with corresponding p-values and ICER (€/LYG). None of the differences of mean survival over time 1 to 6 were significant. Alternatively, the results of weighted long-rank tests did not show significant differences between survival curves (P = 0.32; P = 0.63; P = 0.11). On the other hand, the late-weighted log-rank test (Fleming-Harrington [32]), with p = 0 and q > 0 was significant (P < 0.05), indicating that the treatment group had a significantly better survival curve when adjusting for late effect. This corresponded to an ICER of -€31.97/LYG 6 years after randomization.

**Table 3:** Cost-effectiveness analysis after 6-year follow-up time post-randomization

Years	Mean OS [T (yr)]	Mean OS [C (yr)]	Difference (yr)	P-value	ICER (€/LYG)
1	0.863	0.885	− 0.022	0.57	407.27
2	1.504	1.581	− 0.077	0.47	112.43
3	2.077	2.134	− 0.058	0.74	144.21
4	2.627	2.603	0.025	0.91	− 323.26
5	3.153	3.025	0.129	0.68	− 60.53
6	3.636	3.400	0.236	0.54	− 31.97

Discount rate = 3.5%; Incremental costs = -€8.96; ICER = Incremental cost/LYG; Mean OS = mean survival at a given point calculated as the area under Kaplan–Meier curves

*T* treatment, *C* control, *Yr* year

## Discussion

The results of this trial based economic evaluation show that systemic ONS together with nutritional counseling is both less costly and more effective than nutritional counseling alone, with an ICER if -€3,277/QALY (-€8.96/0.0027QALY). There was, however, high uncertainty around this estimate and the results of the PSA revealed a 53.8% probability of this intervention of being cost-effective at a cost-effectiveness threshold of €30,000/QALY. As the Population EVPI was 8.6 million, it is probably worth recommending performing additional research to resolve the uncertainty currently observed. The latter recommendation is in line with the last American Society for Parenteral and Enteral Nutrition (ASPEN) Value Project Task Force report, suggesting that producing additional high-quality research could generate new insights surrounding the considerable benefits of optimal nutrition care [33].

Systematic nutritional supplementation has shown beneficial effects on a variety of clinical outcomes, and is particularly important in HNC patients, who are at high risk of malnutrition [4–7]. However, we know little about its economic consequences, whose profile is often tied to the type of healthcare setting, nutritional intervention and patients’ group [34]. Economic evaluations of ONS have been conducted in older malnourished hospitalized patients in the USA [35], the Netherlands [36] and Spain [37], admitted with benign gastrointestinal disease [38], hip fracture [39] or for lower gastrointestinal tract surgery [40].

In addition, Elia et al. [1] conducted a systematic review of the cost and cost effectiveness of ONS administered in the hospital setting and showed that the use of ONS compared to “no ONS" or routine care can produce a range of beneficial clinical outcomes, including reduced complications, reduced mortality, more QALY, and reduced length of hospital stay coupled with reduced costs, although only some of the studies showed significant differences. Moreover, Seguy et al. [41] showed that compliance to ONS decreases the risk of hospitalization in malnourished older adults without extra health care cost in France. Direct comparison of this economic evaluation with the above-mentioned studies is difficult because of different patients, diseases, comparators, and healthcare systems.

A strength of this study is that the use of ONS was assessed in a context of appropriate nutritional care as both groups received nutritional counseling [18]. This study has however some limitations. First, it was a single-center study limited to HNC patients, thus transferability of the economic results to other context and populations should be made cautiously. Second, the sample size was determined based on the primary endpoint change in body weight at the end of RT, and not on the cost-effectiveness measures. Third, only direct medical costs were considered, thus preventing to adopt a societal perspective, which is often advocated for making optimal societal decisions [42–45], particularly in the Italian context, where the NHS is a universally accessible service, largely funded through national and regional taxation and thus citizens/taxpayers are interested in an optimal allocation of public resources across sectors.

Interestingly, in the analysis up to 6 years follow-up, we found a significantly better survival in the treatment group when adjusting for late effect. One could question the validity of the late test results if the test was chosen after inspection of the survival curves. Several authors [46–49] have proposed versatile tests using different combinations of weighted log-rank statistics, which allow to test equality of survival curves without making a priori assumptions about the shapes of the survival curves. In this study the most recent versatile test proposed by Karrison [49] was adopted, which uses the maximum of the log-rank (p = 0, q = 0), early-emphasis (p = 1, q = 0), and late-emphasis (p = 0, q = 2) tests as the test statistic and returns a borderline significance (P = 0.065). For this long-term follow up analysis, data were not available to estimate the healthcare resources beyond the end of the study. However, given that during the 5-months intervention the costs were slightly higher in the control group, it would be expected that these would continue to be somewhat higher than in treatment group. Consequently, to assume that there were no further differences in the use of resources between both groups 6 years after the beginning of the intervention can be considered conservative.

## Conclusion

Early ONS provision in combination with nutritional counseling to all head and neck cancer patients treated with radiotherapy provides some impact on quality of cancer care at no significant extra costs from an Italian health care service perspective. National or regional decision-making bodies may refer to this evidence in order to make decisions on whether this intervention should be covered and become an integral part of head and neck cancer care. Trends of potential savings and long-term survival advantage associated with the intervention, should be confirmed in studies with longer follow-up and larger sample size.

## Supplementary Information

**Additional file 1**: **Table S1**. Distribution and parameters used for the Probabilistic Sensitivity Analysis. **Figure S1**. Expected Incremental Benefit (on the left) and Expected Value of Perfect Information (on the right). **Figure S2**. PSA results: Cost Effectiveness Plane (on the left) at a WTP=€30,000/QALY and Acceptability Curve (on the right).

## Data Availability

Individual patient data and scripts are available from the authors upon request.
