# Supplementary material for: Cost-effectiveness analysis of oral nutritional supplements with nutritional counselling in head and neck cancer patients undergoing radiotherapy
Source: Cost Eff Resour Alloc. 2021 Jun 15;19:35. doi: 10.1186/s12962-021-00291-7 (PMC8207624; doi:10.1186/s12962-021-00291-7)

**Supplementary Table 1.** Distribution and parameters used for the Probabilistic Sensitivity Analysis

|  | **Distribution** | **AIC** | **Shape** | **Scale** |
| --- | --- | --- | --- | --- |
| Costs treatment | Gamma | 1206.3 | 2.51 | 393.08 |
| Costs control | Weibull | 955.7 | 0.31 | 131.56 |
| 0.417-QALY treatment | Weibull | -178.5 | 1.47 | 0.14 |
| 0.417-QALY control | Weibull | -185.1 | 1.53 | 0.14 |

**Supplementary Figure 1.** Expected Incremental Benefit (on the left) and Expected Value of Perfect Information (on the right)


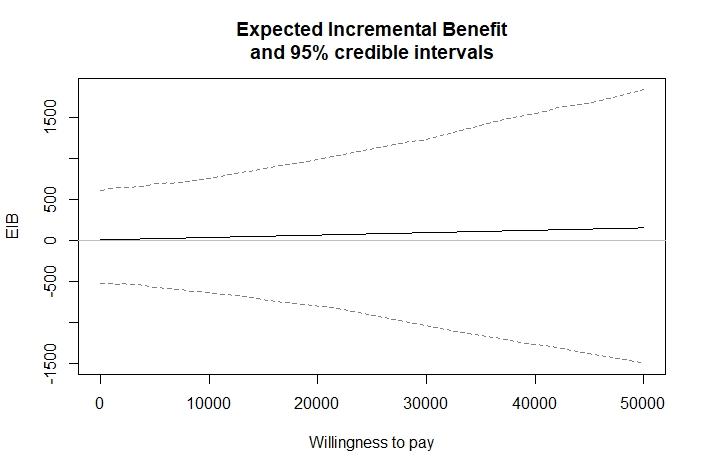

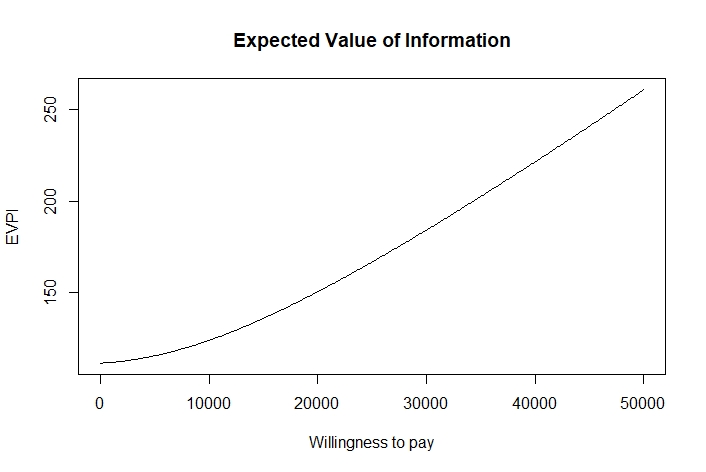


**Supplementary Figure 2**. PSA results: Cost Effectiveness Plane (on the left) at a WTP=€30,000/QALY and Acceptability Curve (on the right)


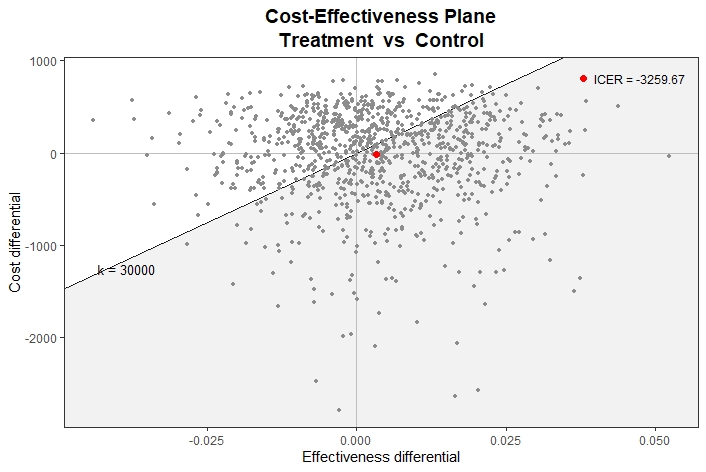

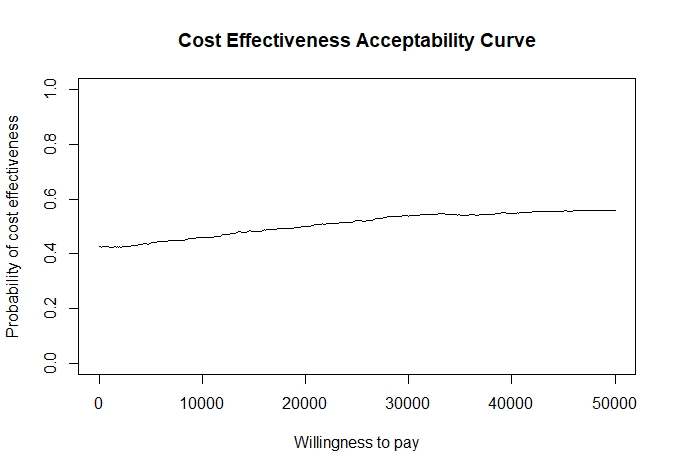

Supplement: Supplementary file 1 — Additional file 1: Table S1. Distribution and parameters used for the Probabilistic Sensitivity Analysis. Figure S1. Expected Incremental Benefit (on the left) and Expected Value of Perfect Information (on the right). Figure S2. PSA results: Cost Effectiveness Plane (on the left) at a WTP=€30,000/QALY and Acceptability Curve (on the right). [file 12962_2021_291_MOESM1_ESM.docx]
